# Investigation of a model for evaluating cognitive decline from facial photographs using AI

**DOI:** 10.1111/ggi.14793

**Published:** 2024-01-02

**Authors:** Yumi Umeda‐Kameyama, Masashi Kameyama, Taro Kojima, Tomoki Tanaka, Katsuya Iijima, Sumito Ogawa, Tomomichi Iizuka, Masahiro Akishita

**Affiliations:** ^1^ Dementia Center The University of Tokyo Hospital Tokyo Japan; ^2^ Department of Geriatric Medicine The University of Tokyo Tokyo Japan; ^3^ Tokyo Metropolitan Institute for Geriatrics and Gerontology Tokyo Japan; ^4^ Institute of Gerontology, The University of Tokyo Tokyo Japan; ^5^ Institute for Future Initiatives, The University of Tokyo Tokyo Japan; ^6^ Center for Dementia Fukujuji Hospital Kiyose Japan

Dear Editor,

Japan has more than 5 million people with dementia, and if 4 million people with mild cognitive impairment are included, one in four older adults is in a state of declining cognitive function. Early detection of cognitive decline can prevent the onset of dementia. Developing a less invasive, inexpensive and simple dementia screening tool is urgently required.

Perceived age is more strongly correlated with cognitive function compared with chronological age.^1^ Thus, it was postulated that cognitive decline might be expressed in a patient's face. Then, we carried out a study using artificial intelligence (AI), which can likely determine more details compared with human judgment. When deep learning was used to learn facial photographs of patients with Alzheimer's disease and normal facial photographs, and a classification task was attempted using the test images, the correct answer rate was 92.6%, and the area under the curve was 0.97.

The scores calculated by the AI model correlated significantly more strongly with cognitive decline than with chronological age.^2^

While perceived age can be a useful biomarker in the geriatric field, it requires more than 10 people to judge it. AI that determines age from facial photos is already in use, and for this study, we opted to utilize the widely used Microsoft Azure Application Programming Interface. We aimed to determine if AI can easily produce reliable perceived age.

The Alzheimer's disease group (121 people), which was used for the previous study,^2^ was also used in the present study. We evaluated their faces using the Microsoft Azure face Application Programming Interface.

The AI Azure age showed significant correlation with chronological age (*r* = 0.480, *P* = 2.53 × 10^−8^) and human‐judged perceived age” (*r* = 0.791, *P* = 3.88 × 10^−27^; Fig. S1); however, it failed to show a significantly stronger correlation with the Mini‐Mental State Examination than chronological age (Table 1) in men, women and all participants, contrary to human‐judged perceived age. Furthermore, although human perceived age showed a stronger correlation with the Vitality Index^3^ than chronological age,^1^ AI Azure age failed to show superiority (Data S2). To test the hypothesis that a higher population might overcome the failure of AI Azure age, we tried 638 faces; however, AI Azure age failed to show the superiority to chronological age (Data S3). We tested the relationship between “sadness” detected by Azure and GDS15. However, the analysis of sadness also failed to detect depression (Data S4, Fig. S2).

**Table 1:** Mini‐Mental State Examination and artificial intelligence Azure age

MMSE		*r*	*P*
Total	Chronological	−0.166	0.0688
(*n* = 121)	Azure	−0.186	0.0416
	Difference		0.833
Male	Chronological	−0.200	0.187
(*n* = 45)	Azure	−0.184	0.226
	Difference		0.923
Female	Chronological	−0.145	0.212
(*n* = 76)	Azure	−0.206	0.0749
	Difference		0.572

*Note*: Difference refers to a test of the difference between correlated correlation coefficients, known as Steiger's test.

The failure to show the superiority of AI‐perceived age over human‐judged perceived age might be attributable to the weakness of the Azure system in older people and Asian ethnicity. The AI Azure age perceived age to be approximately 20 years younger than chronological age, and the maximum value was 74 years. Emotion judged by AI was reported to be biased by population due to bias in training data.^4^

To determine the perceived age of older individuals as a useful biomarker, AI trained on a substantial amount of training data from older individuals would likely be necessary.

## Disclosure statement

The authors declare no conflict of interest.

## Supporting information


**DATA S1.** Supporting Information.

## Data Availability

It is strongly prohibited by the Ethics Committee to provide the face photos externally.
