# Supplementary material for: Investigation of a model for evaluating cognitive decline from facial photographs using AI
Source: Geriatr Gerontol Int. 2024 Jan 2;24(Suppl 1):393–4. doi: 10.1111/ggi.14793 (PMC11503600; doi:10.1111/ggi.14793)
Supplement: Supplementary file 1 — DATA S1. Supporting Information. [file GGI-24-393-s001.pdf]

LETTER SUPPORTING INFORMATION

Dementia

Investigation of a Model for Evaluating Cognitive Decline from Facial Photographs Using AI

Yumi Umeda-Kameyama[1,2] | Masashi Kameyama[3] | Taro Kojima[2] | Tomoki Tanaka[4] | Katsuya Iijima[4,5] | Sumito Ogawa[2] | Tomomichi Iizuka[6] | Masahiro Akishita[2]

[1]Dementia Center, The University of Tokyo Hospital, Tokyo, 113-8655, Japan

[2]Department of Geriatric Medicine, The University of Tokyo, Tokyo, 113-8655, Japan

[3]AI & Theoretical Image Processing, Research Team for Neuroimaging, Tokyo Metropolitan Institute for Geriatrics and Gerontology, Tokyo, 173-0015, Japan

[4]Institute of Gerontology, The University of Tokyo, Tokyo, 113-0033, Japan

[5]Institute for Future Initiatives, The University of Tokyo, Tokyo, 113-0033, Japan

[6]Center for Dementia Fukujuji Hospital, Kiyose, Tokyo, 204-8522, Japan

Correspondence
Dr. Yumi Umeda-Kameyama, MD, PhD, Dementia Center, The University of Tokyo Hospital, Tokyo, Postal Code, Japan
Email: yumeda-tky@umin.ac.jp

Funding information

In this SUPPORTING INFORMATION, we provide Correlation between AI Azure age and chronological age / perceived age, analysis of Vitality Index, MMSE evaluation with more population and Evaluation of sadness.

1 | CORRELATION BETWEEN AI AZURE AGE AND CHRONOLOGICAL AGE / PERCEIVED AGE

AI azure age showed significant correlation with chronological age (male: $r = 0.408, p = 0.00535$; female: $r = 0.563, p = 1.22 \times 10^{-7}$, all: $r = 0.480, p = 2.53 \times 10^{-8}$) and human perceived age (male: $r = 0.665, p = 6.17 \times 10^{-7}$, female

$r = 0.815, p : 3.56 \times 10^{-19}$, all $r = 0.791, p = 3.88 \times 10^{-27}$). The difference in the two correlation coefficients were significant (male: $p = 0.0268$, female: $p = 0.000101$, all: $p = 8.82 \times 10^{-8}$). Scatter plots are shown in SUPPORTING FIGURE 1.

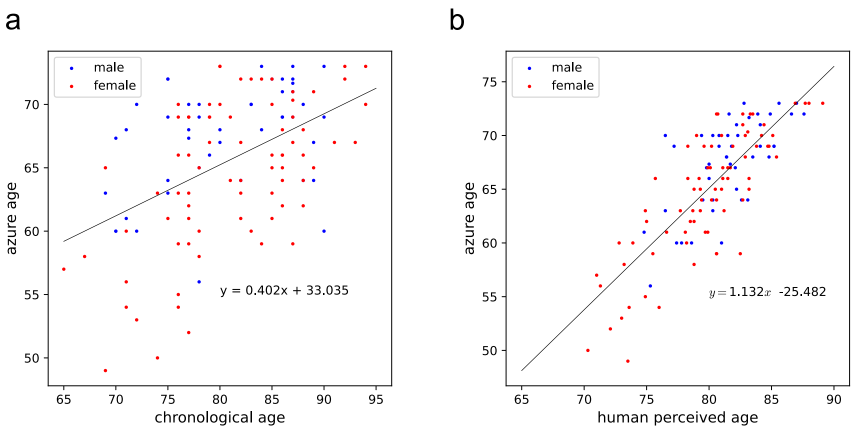

SUPPORTING FIGURE 1 Scatter plots between AI azure age and chronological age (a) / perceived age (b). Black lines are regression lines. (a: $y = 0.4025x + 33.03$; b: $y = 1.132x - 25.48$)

2 | VITALITY INDEX

Correlation between Vitaliy Index[1] and chronological / AI azure age is shown in SUPPORTING TABLE 1. Our previous study[2] showed that human perceived age showed significantly stronger correlation with vitality index than chronological age in female ($p = 0.0122$) and total ($p = 0.00741$) populations. However, AI azure age failed to show the superiority to chronological age.

SUPPORTING TABLE 1 Correlation between Vitaliy Index and chronological / AI azure age

Vitality Index		*r*	*p*
total	chrological	−0.276	0.00314
(*n*=113)	azure	−0.287	0.00207
	difference		0.902
male	chrological	−0.203	0.208
(*n*=40)	azure	−0.0630	0.699
	difference		0.430
female	chrological	−0.320	0.00573
(*n*=73)	azure	−0.369	0.00130
	difference		0.637

3 | MMSE EVALUATION WITH MORE POPULATION

MMSE analysis with AD patients from the University of Tokyo Hospital failed show the superiority of AI azure age over chronological age (Table 1). To determine that the failure is attibutable to the small sample size, normal participants from Kashiwa cohort study (n=117) and normal and AD participants from Fukujuji Hospital (n=400) as well as AD participants from the University of Tokyo were included in this analysis as their MMSE and photographs were available. AI azure age failed to show the superiority over chronological age, again (SUPPORTING TABLE 2).

SUPPORTING TABLE 2 Correlation between MMSE and chronological / AI azure age with larger population

MMSE		r	p
total	chrological	−0.256	5.62×10^{-11}
(n=638)	azure	−0.215	4.33×10^{-8}
	difference		0.283
male	chrological	−0.273	5.69×10^{-8}
(n=382)	azure	−0.262	1.96×10^{-7}
	difference		0.817
female	chrological	−0.232	0.000177
(n=256)	azure	−0.143	0.0220
	difference		0.132

4 | EVALUATION OF DEPRESSION

Microsoft Azure AI Face recognizes various characteristics of faces including age, sex, happiness and sadness. We hypothesized “sadness” that azure face API recognizes might be useful to detect depression. However, “sadness” did not show correlation with GDS15 (SUPPORTING FIGURE 2). Furthermore, any of the characteristics of azure face API did not show high correlation with GDS-15 (azure age: 0.037, anger: −0.045, contempt: 0.000, disgust: −0.163, fear: −0.143, happiness: 0.027, neutral: −0.014, sadness: 0.020, surprise: −0.126, bald: −0.061).

references

[1] Toba K, Nakai R, Akishita M, Iijima S, Nishinaga M, Mizoguchi T, et al. Vitality Index as a useful tool to assess elderly with dementia. Geriatrics & gerontology international. 2002;**2**(1):23–29.

[2] Umeda-Kameyama Y, Kameyama M, Kojima T, Ishii M, Kidana K, Yakabe M, et al. Cognitive function has a stronger correlation with perceived age than with chronological age. Geriatrics & Gerontology International. 2020;**20**(8):779–784.

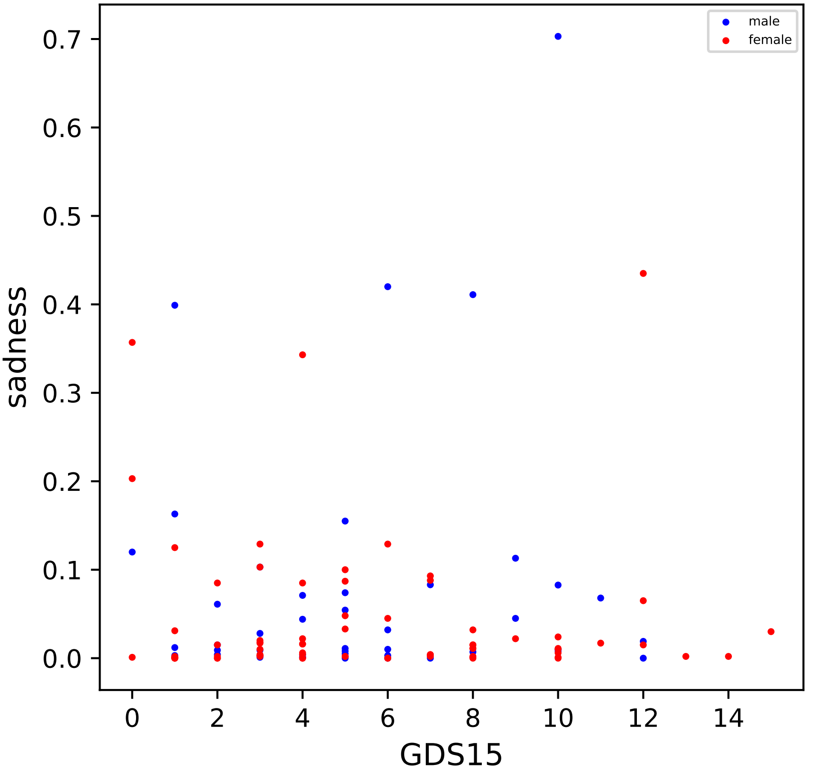

SUPPORTING FIGURE 2 Relationship between "sadness" evaluated by azure and GDS15. Pearson's correlation coefficient (r) was 0.020.
